# Correlation of early-phase β-amyloid positron-emission-tomography and neuropsychological testing in patients with Alzheimer’s disease

**DOI:** 10.1007/s00259-025-07175-5

**Published:** 2025-02-28

**Authors:** Friederike Völter, Sebastian Eckenweber, Maximilian Scheifele, Florian Eckenweber, Fabian Hirsch, Nicolai Franzmeier, Annika Kreuzer, Maria Griessl, Anna Steward, Daniel Janowitz, Carla Palleis, Alexander Bernhardt, Jonathan Vöglein, Anna Stockbauer, Boris-Stephan Rauchmann, Florian Schöberl, Elisabeth Wlasich, Katharina Buerger, Olivia Wagemann, Robert Perneczky, Endy Weidinger, Günter Höglinger, Johannes Levin, Matthias Brendel, Sonja Schönecker

**Affiliations:** 1 Department of Nuclear Medicine, University Hospital of Munich, LMU Munich, Munich, Germany; 2 Department of Internal Medicine IV, University Hospital of Munich, LMU Munich, Munich, Germany; 3 Institute for Stroke and Dementia Research (ISD), Munich, Germany; 4 Munich Cluster for Systems Neurology (SyNergy), Munich, Germany; 5 Institute of Neuroscience and Physiology, Department of Psychiatry and Neurochemistry, The Sahlgrenska Academy, University of Gothenburg, Mölndal and Gothenburg, Sweden; 6 Department of Neurology, LMU University Hospital, LMU Munich, Munich, Germany; 7 German Center for Neurodegenerative Diseases (DZNE), Munich, Germany; 8 Department of Neuroradiology, University Hospital, Ludwig Maximilian University of Munich, Munich, Germany

**Keywords:** Early-phase, β-amyloid, PET/CT, Alzheimer’s disease, Neurodegenerative diseases, Neuropsychological testing, MMSE, CERAD

## Abstract

**Purpose:**

Clinical staging in individuals with Alzheimer’s disease (AD) typically relies on neuropsychological testing. Recognizing the imperative for an objective measure of clinical AD staging, regional perfusion in early-phase β-amyloid-PET may aid as a cost-efficient index for the assessment of neurodegeneration severity in patients with Alzheimer’s disease.

**Methods:**

Regional perfusion deficits in early-phase β-amyloid-PET as well as neuropsychological testing (max. 90 days delay) were evaluated in 82 patients with biologically defined AD according to the ATN classification. In reference to the Braak staging system patients were classified into the groups stage^0^, stage^I−II+^, stage^I−IV+^, stage^I−VI+,^ and stage^atypical+^ according to regional perfusion deficits in regions of interest (ROIs) published by the Alzheimer’s Disease Neuroimaging Initiative. Multiple regression analysis controlling for age, gender, and education was used to evaluate the association of regional z-scores on perfusion-phase PET with clinical scores for all patients and with annual decline of cognitive performance in 23 patients with follow-up data.

**Results:**

Patients classified as stage^0^ and stage^I−II+^ demonstrated significantly superior neuropsychological performance compared to those classified as stage^I−IV+^ and stage^I−VI+^. Lower cognitive performance was associated with decreased perfusion in early-phase β-amyloid-PET globally and regionally, with the most pronounced association identified in the left temporal lobe. Mean z-scores on early-phase PET in temporal and parietal regions offered a robust prediction of future annual decline in MMSE and sum scores of the CERAD-Plus (Consortium to Establish a Registry for Alzheimer’s Disease) test battery.

**Conclusion:**

Regional and global perfusion deficits in early-phase β-amyloid-PET can serve as an objective index of neurodegeneration severity and may act as prognostic markers of future cognitive decline in AD.

**Supplementary Information:**

The online version contains supplementary material available at 10.1007/s00259-025-07175-5.

## Introduction

For the in vivo diagnosis of Alzheimer’s disease (AD), the “A/T/N” classification system, focusing on β-amyloid deposition (A), pathologic tau (T), and neuronal injury (N), is widely accepted for the biological definition of the disease [1]. These ATN criteria can be evaluated using cerebrospinal fluid (CSF), plasma, and imaging biomarkers. Among imaging biomarkers, FDG-PET is commonly used to assess neuronal injury [1–3]. Regional neuronal injury measured by FDG-PET has been associated with neurocognitive performance on clinical tests; however, its reliability as a cognitive marker is limited by variations in cognitive resilience and unmeasured copathologies [2, 4, 5]. Beyond FDG-PET, early-phase β-amyloid-PET imaging provides an alternative means of assessing neuronal injury, utilizing tracer extraction from blood as a perfusion surrogate [6–10]. The strong correlation between regional uptake in the perfusion phase of beta-amyloid PET and regional neuronal damage in FDG-PET is likely driven by neurovascular coupling of cerebral blood flow and metabolic demand [11–13].

A dual-phase acquisition protocol in β-amyloid-PET imaging enables not only the reliable detection of cerebral β-amyloid plaques (i.e., A) [14–19] but also the assessment of neuronal injury (i.e., N). The combination of perfusion-surrogate imaging and β-amyloid plaque detection within a single scan reduces the need for multiple imaging modalities and improving efficiency for patients, physicians, and the healthcare system.

While imaging biomarkers provide objective measures of AD pathology, their clinical relevance must be validated against neurocognitive performance. The clinical severity of AD is commonly screened using the Mini-Mental state examination (MMSE) [20, 21]. To further assess cognitive decline in more detail and thus confirm suspicion of cognitive decline by MMSE, more advanced neuropsychological test batteries such as the CERAD-Plus test battery are available [22]. For therapeutic studies, a crucial necessity arises for objective biomarkers that precisely correlate with disease severity, progression rate, and underlying pathology. There is limited research on the association between regional perfusion deficit in perfusion-phase β-amyloid-PET and clinical severity of dementia [23]. By comparing regional perfusion deficits, current neuropsychological test results, and future annual cognitive decline, we aimed to investigate the agreement between early-phase β-amyloid-PET and cognitive performance of patients with AD. Additionally, we explored whether perfusion-phase β-amyloid PET can serve as a biomarker for in vivo staging of disease severity, potentially enhancing diagnostic accuracy and disease monitoring.

## Methods

### Patient acquisition

All patients receiving dual-phase β-amyloid-PET between September 2013 and October 2021 were evaluated for inclusion in the study. Inclusion criteria were amyloid-positivity on visual inspection of the PET scan [1] as well as an interval of less than 90 days between PET scan and neuropsychological testing (e.g. MMSE and CERAD-PLUS test battery). Patients with atypical AD or mixed pathology were excluded.

### Clinical evaluation

Clinical severity of dementia was assessed using the MMSE and CERAD-PLUS test battery by neuropsychologists and physicians of the Institute for Stroke and Dementia Research, the Department of Psychiatry and Psychotherapy, and the Department of Neurology at LMU University Hospital, LMU Munich, Munich, Germany [20, 22]. In cases, when MMSE was not available, Montreal Cognitive Assessment Scores were used.

### Radiosynthesis and image acquisition

The radiosynthesis of [^18^F]flutemetamol and [^18^F]florbetaben was performed as described previously. After semipreparative high-performance liquid chromatography, radiochemical purity was > 97% [24]. PET images were acquired with a Biograph 64 or a Siemens mCT PET/CT scanner (Siemens Healthineers, Erlangen, Germany) at the Department of Nuclear Medicine, LMU University Hospital, LMU Munich, Germany. The mean injected dose of [^18^F]florbetaben (*n* = 36) was 286 MBq (± 52) / 7.73 mCi (± 1.41). The mean injected dose of [^18^F]flutemetamol (*n* = 47) was 177 MBq (± 22) / 4.78 mCi (± 0.59). The perfusion-weighted scan was acquired 0–10 min post-injection as a single frame [6].

### Image processing

Brains were parcellated into 47 regions using the automated anatomical labeling (AAL) atlas with Hermia Neurology Package Version 6.1.4 (Brass) (Hermes Medical Solutions, Stockholm) [25]. Regional tracer activity of single brain regions was normalized to the global mean activity of the patient and related to a built-in normal cohort of FDG-PET (z-score) as decribed previously [7]. Voxel-based volume-weighted mean z-scores were calculated for all cortical brain lobes of both sides. Furthermore, we investigated z-scores in predefined volumes of interest in reference to the Braak stage composite regions of interest published by the Alzheimer’s Disease Neuroimaging Initiative [26]. Specifically, regions I/II were defined as the mesiotemporal lobes. Regions III/IV included the mesio-occipital lobes, the mid and inferior temporal cortex, the temporal pole, the anterior and posterior cingulate and the insular cortex. Regions V/VI encompassed the orbitofrontal cortex, the frontal lateral cortex, the frontal medial cortex, the superior temporal cortex, the occipital cortex, the superior and inferior parietal cortex, the precuneus and the post- and precentral cortex.

### Regional staging with early-phase PET data

A ratio was calculated by dividing the voxel-weighted mean z-score of regions I/II, III/IV and V/VI of the patient’s PET by the voxel-weighted mean z-score of the respective regions in patients with an MMSE-score above 27. Based on previous studies evaluating PET-based classification of disease severity, a cut-off of -1.3 was used to determine whether the perfusion in a volume of interest (VOI) was abnormal [27]. Patients were classified as stage^I−II+^ when only the regions I/II surpassed the cut-off of 1.3. When the regions I/II as well as the regions III/IV surpassed the cut-off, but not regions V/VI, patients were classified as stage^I−IV+^. When the regions I/II, III/IV, and V/VI all surpassed the cut-off, patients were staged stage^I−VI+^. Patients with no region surpassing the threshold were labelled stage^0^. Patients deviating from this staging scheme were labelled stage^atypical+^ [27].

### Statistics

Patients were divided into 5 groups according to their MMSE score (group I: MMSE-score 28–30, group II: MMSE-score 24–27, group III: MMSE-score 18–23, group IV: MMSE-score 10–17, group V: MMSE-score < 9). For three patients, MMSE scores were not available. Montreal Cognitive Assessment scores were converted to an equivalent MMSE score by Lawton et al. [28, 29]. Normal distribution was tested using the Shapiro-Wilk test. Quantitative values were reported with mean **±** standard deviation when normally distributed and with median and interquartile range when not normally distributed. For multi-comparison testing volume-weighted mean z-scores of all groups were compared by Kruskal-Wallis-test or ANOVA followed by Tukey’s multiple comparisons test.

Clinical follow-up data were available in 23 patients. To calculate percentage annual decline the decrease of MMSE scores and CERAD sum scores was divided by the study interval in years.

Volume-weighted mean z-scores of all brain lobes and single brain regions as well as volume-weighted mean z-scores of regions I and II, regions III and IV, as well as regions V and VI were compared with clinical test scores and annual percentage decline by applying multiple linear regression correcting for age, gender and years of education.

## Results

### Study cohort

**Fig. 1:**
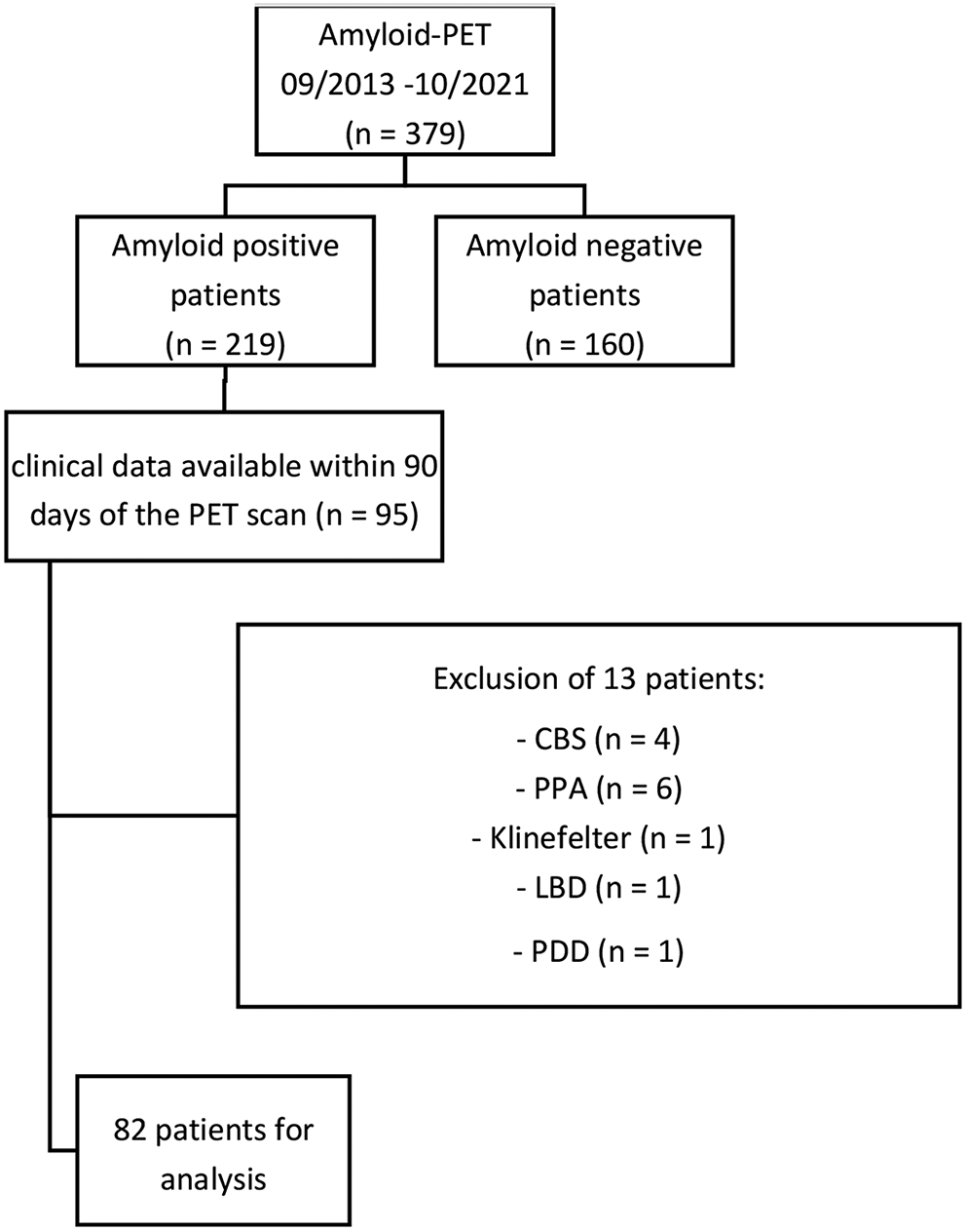
Flow chart of patient selection

379 patients were screened for inclusion in the study. After applying the inclusion and exclusion criteria described, a total of 82 patients were included in the study. Specific reasons for inclusion and exclusion of patients are shown in a flowchart in Fig. 1. Table 1 presents the neuropsychological test scores obtained from MMSE testing and the CERAD Plus test battery, categorizing patients into groups according to their MMSE test scores. No significant differences were observed in age, sex, and years of education among patient groups with varying clinical severity. Patients with decreased MMSE scores also showed significantly lower test scores in the CERAD Plus test battery evaluating the total score and most of the CERAD Plus subtests. Post-hoc multiple comparisons tests are added in supplementary Table 1.

**Table 1:** Patient cohort with categorization into groups based on MMSE scores

	All patients	MMSE 28–30	MMSE 24–27	MMSE 18–23	MMSE 10–17	*p* ^1^
**Number of patients**	82	14	36	21	11	
**Mean age**	71.3 (± 8.8)	70.3 (± 8.4)	71.8 (± 6.9)	71.4 (± 8.2)	73.1 (± 8.6)	0.745
**Sex (female/male)**	(40/42)	(5/9)	(18/18)	(10/11)	(7/4)	0.557
**Years of education**	13.4 (± 3.3)	14.6 (± 3.7)	13.2 (± 2.9)	12.6 (± 2.8)	13.1 (± 4.6)	0.366
**MMSE score**	23.4 (± 4.8)	28.8 (± 0.9)	25.2 (± 1.0)	21.7 (± 1.3)	13.6 (± 2.6)	**< 0.0001**
**CERAD test battery**						
**Total score**	59.3 (± 12.3)	68.9 (± 8.6)	61.7 (± 12.0)	52.3 (± 9.3)	43.7 (± 10.3)	**< 0.0001**
Word list learning	11.2 (± 5.5)	14.5 (± 5.2)	11.8 (± 5.6)	11.1 (± 3.3)	4.7 (± 4.6)	**< 0.0001**
Word list recall	2.9 (± 2.1)	5.2 (± 1.8)	3.1 (± 1.8)	1.8 (± 1.5)	0.5 (± 0.9)	**< 0.0001**
Word list intrusions	2.7 (± 3.7)	1.4 (± 1.4)	2.7 (± 3.5)	3.8 (± 4.9)	1.7 (± 2.2)	0.207
Word list recognition	8.5 (± 1.9)	9.5 (± 0.7)	8.4 (± 1.9)	8.1 (± 2.2)	7.9 (± 2.2)	0.121
Word list discriminability	6.6 (± 3.8)	7.4 (± 4.0)	6.2 (± 4.0)	7.3 (± 3.5)	5.3 (± 1.5)	0.382
Figure drawing	9.2 (± 2.5)	10.9 (± 0.3)	9.7 (± 1.9)	7.7 (± 2.8)	7.4 (± 3.1)	**< 0.0001**
Figure recall	4.3 (± 3.0)	7.2 (± 3.0)	4.7 (± 2.4)	2.9 (± 2.1)	0.7 (± 1.2)	**< 0.0001**
Semantic verbal fluency	13.8 (± 5.0)	14.5 (± 4.8)	15.5 (± 4.3)	12.3 (± 3.9)	9.0 (± 6.1)	**< 0.001**
Phonematic verbal fluency	9.8 (± 4.3)	11.3 (± 4.0)	10.3 (± 4.4)	8.7 (± 3.4)	6.7 (± 3.9)	**0.027**
Boston naming test	12.8 (± 2.5)	13.6 (± 1.5)	13.4 (± 1.9)	12.3 (± 1.9)	10.2 (± 3.9)	**< 0.001**
Trail making test A	76.0 (± 39.7)	57.6 (± 22.5)	74.5 (± 36.9)	84.2 (± 31.5)	121.8 (± 73.6)	0.004

^1^Analysis of variance between displayed patient groups. The significance level was adjusted with Bonferroni correction to α = 0.003215. Significant p-values are displayed bold

### Data driven multilinear regression of regional perfusion deficits and cognitive performance in patients with AD

**Fig. 2:**
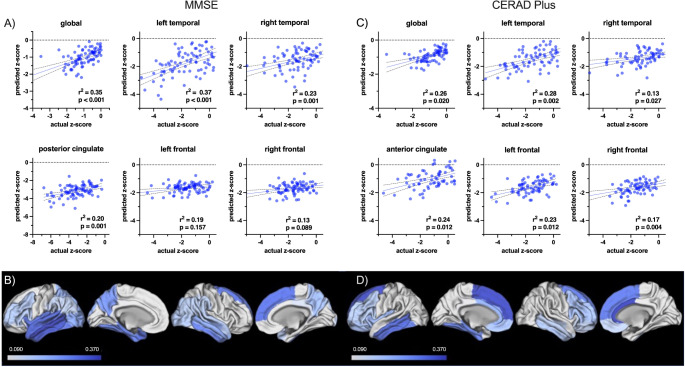
Multilinear regression of neuropsychological test results and regional perfusion deficits corrected for age, sex and years of education. (**A**) and (**B**) partial correlation (r^2^) of regional perfusion deficits and MMSE scores, (**C**) and (**D**) partial correlation (r^2^) of regional perfusion deficits and CERAD Plus sum scores

Multiple linear regression corrected for age, sex, and education showed a moderate association between MMSE scores and mean z-scores of both temporal lobes (r^2^ = 0.37, *p* < 0.0001) and the global perfusion deficit (r^2^ = 0.35, *p* < 0.001), as well as a weak association with perfusion deficits in the parietal lobes (r^2^ = 0.09–0.14, *p* = 0.012–0.028), the precuneus (r^2^ = 0.15–0.22, *p* < 0.01), and the posterior cingulate cortex (r^2^ = 0.20, *p* = 0.001). CERAD-Plus total score, semantic word fluency, and the Boston naming test showed a moderate association with left frontal lobe, left temporal lobe, and global perfusion deficits (r^2^ = 0.21–0.39, *p* < 0.0001–0.020). Trail making test (TMT) A demonstrated a significant moderate association with perfusion deficits in the temporal lobe, parietal lobe, posterior cingulate cortex (PCC), left precuneus, and occipital lobe as well as with the global perfusion deficit (r^2^ = 0.19–0.41, *p* < 0.0001–0.019). Almost all subtests were more strongly associated with left hemispheric compared to right hemispheric perfusion deficits. Detailed results are displayed in Table 2; Fig. 2.

**Table 2:**
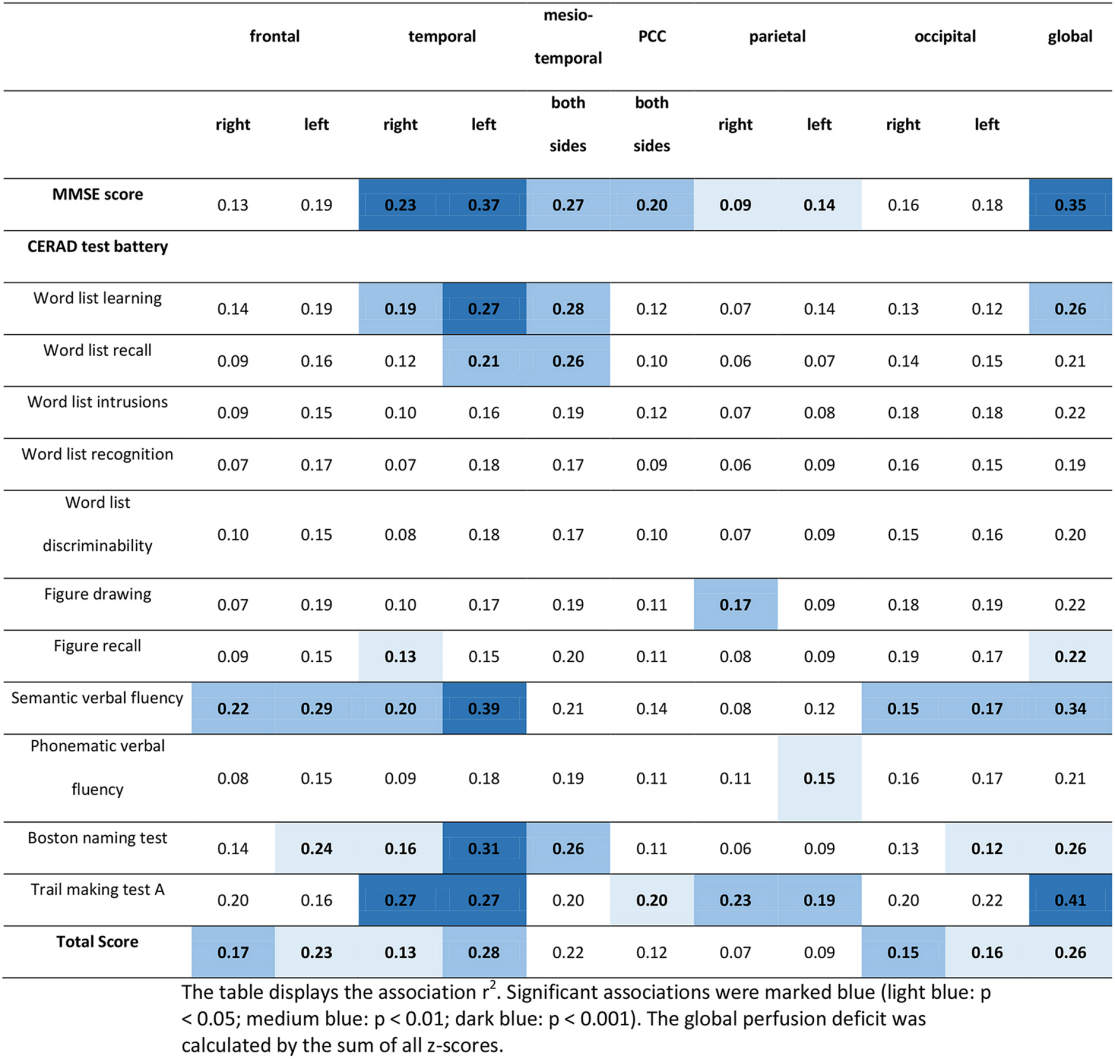
Multilinear regression of neuropsychological test results and regional perfusion deficits correcting for age, sex, and education

### Association between perfusion deficits in predefined regions of interest and cognitive impairment

Multiple linear regression showed a significant association of perfusion deficits in all predefined regions I-VI with MMSE scores (r^2^ = 0.24–0.33; *p* < 0.0001–0.003) and the CERAD-Plus sum score (r^2^ = 0.20–0.27; *p* = 0.006–0.048). Within the CERAD-Plus subtests, the strongest association could be detected between perfusion deficits of regions III/IV and V/VI and the TMT A as well as semantic word fluency (r^2^ = 0.27–0.36; *p* < 0.0001–0.0004). Additionally, there was a significant association between the items word list learning, word list recalling and perfusion deficit in regions I/II and III/IV (r^2^ = 0.22–0.27; *p* = 0,007–0.031) and between the Boston Naming Test and all predefined regions (r^2^ = 0.19–0.26; *p* = 0.006–0.027).

**Fig. 3:**
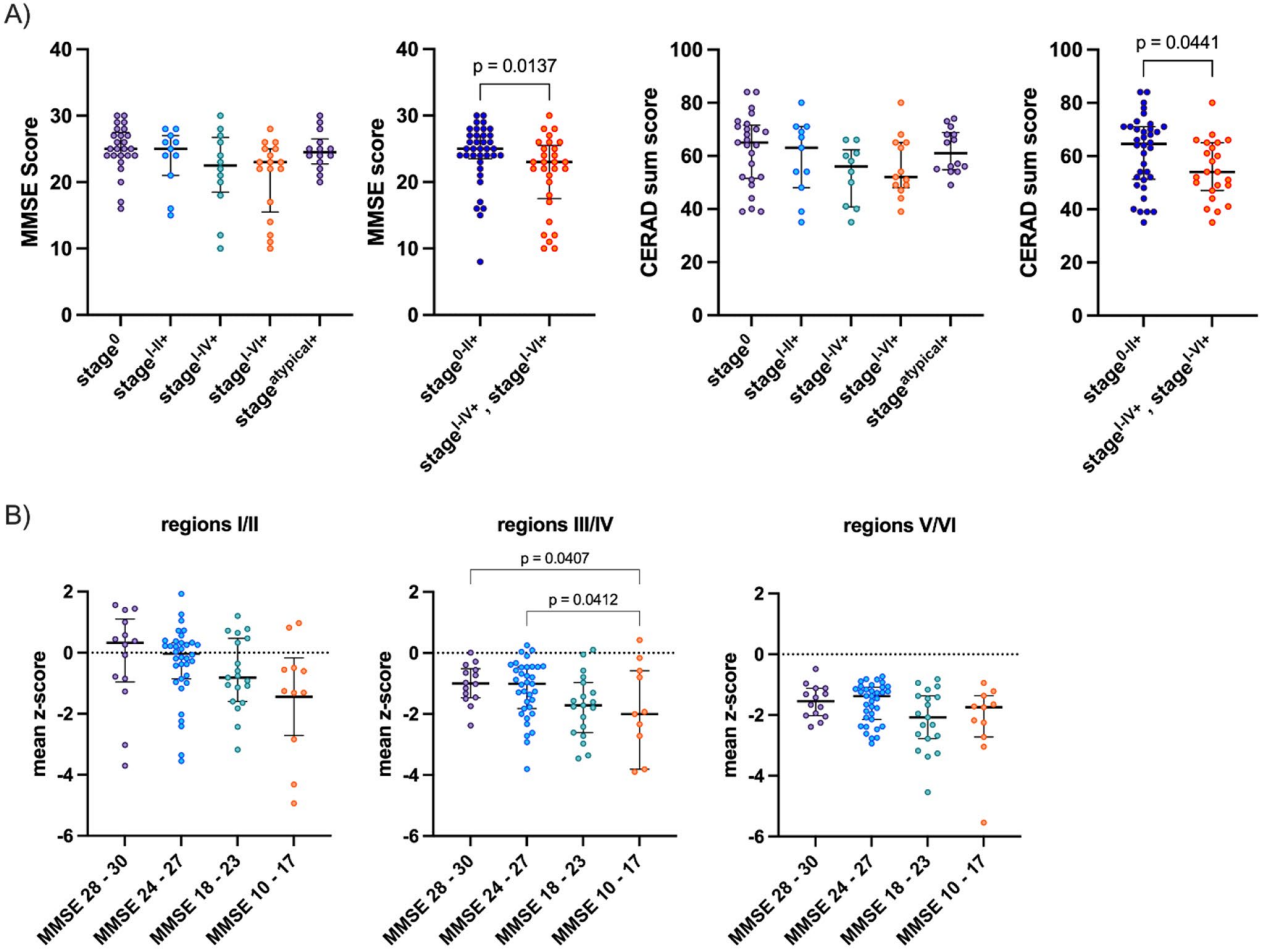
Association between perfusion deficits in predefined regions of interest and neuropsychological testing with MMSE and CERAD-Plus test battery **A**) Cognitive performance in MMSE and CERAD-Plus test battery in patients grouped according to their PET-based stage evaluated with regional perfusion deficits in early-phase β⁠-amyloid-PET. **B**) Perfusion deficits in predefined regions of interest in patients with differing test results in MMSE testing

Patients that were classified as stage^0+^ (*n* = 25) and stage^I−II+^ (*n* = 11) had significantly better cognitive performance than patients that were classified as stage^I−IV+^ (*n* = 13) and stage^I−VI+^ (*n* = 17) when testing with MMSE (*p* = 0.014) or CERAD-Plus test battery (*p* = 0.044) at the time of imaging (Fig. 3A). 16/82 patients (19.5%) were classified as stage^atypical+^.

### Perfusion deficits across clinical stages and PET-based stages in patients with AD

All MMSE-related subgroups of patients with AD showed most severe perfusion deficits in the precuneus, the inferior parietal lobe, the mid temporal lobe, and the posterior cingulate gyrus (supplementary Table 2). With increasing severity of dementia, perfusion deficits measured by z-scores worsened in the described regions, as well as in various other regions like the occipital lobe, the anterior cingulate gyrus, and the insula (supplementary Table 1). In addition, z-scores decreased with increasing clinical severity in all PET-based stage-associated regions. While in the predefined region III/IV the decrease was statistically significant (*p* = 0.02), statistical significance was not reached in regions I/II and V/VI (*p* = 0.092 and 0.125). Perfusion deficits of the predefined stage-associated regions and an analysis of variance between patient groups stratified according to their MMSE score are displayed in Table 3 and in Fig. 3B). Post-hoc multiple comparisons test showed a significant difference of regional hypoperfusion in regions III/IV in patients with MMSE 10–17 compared to patients with MMSE 24–27 and MMSE 28–30 (*p* = 0.041, respectively) (supplementary Table 3).

**Fig. 4:**
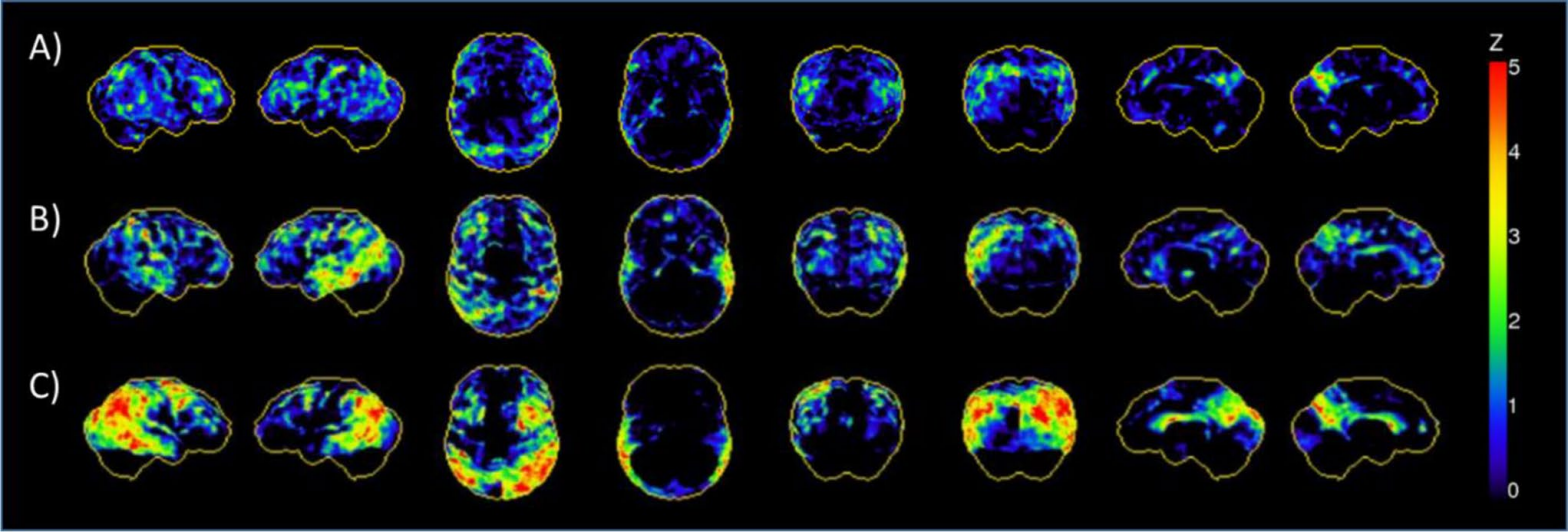
Surface projections (3DSSP) of patients with different PET-based stages **A**) 3DSSP of a patient with subjective cognitive complaints (MMSE 28; CERAD Plus sum score 80), PET-based staging returned stage^I−II+^. **B**) 3DSSP of a patient with mild dementia (MMSE 23; CERAD Plus sum score 39), PET-based staging returned stage^I−IV+^. **C**) 3DSSP of a patient with moderate dementia (MMSE 11; CERAD Plus sum score not assessable), PET-based staging returned stage^I−VI+^

Figure 4 displays surface projections (3DSSP) of three exemplary patients with varying PET-based stages.

**Table 3:** Perfusion deficit in AD-related regions of patient groups stratified according to clinical severity expressed as volume-weighted mean z-score and standard deviation

	MMSE 28–30	MMSE 24–27	MMSE 18–23	MMSE 10–17	*p*
regions I/II	-0.19 ± 1.55	-0.32 ± 1.18	-0.70 ± 1.14	-1.44 ± 1.80	0.092
regions III/IV	-1.01 ± 0.62	-1.20 ± 0.93	-1.74 ± 0.99	-2.29 ± 2.09	**0.020**
regions V/VI	-1.55 ± 0.53	-1.62 ± 0.64	-2.16 ± 0.97	-2.21 ± 1.21	0.125

### Early-phase β-amyloid-PET predicts future annual decline of MMSE test scores and CERAD-Plus test scores

**Fig. 5:**
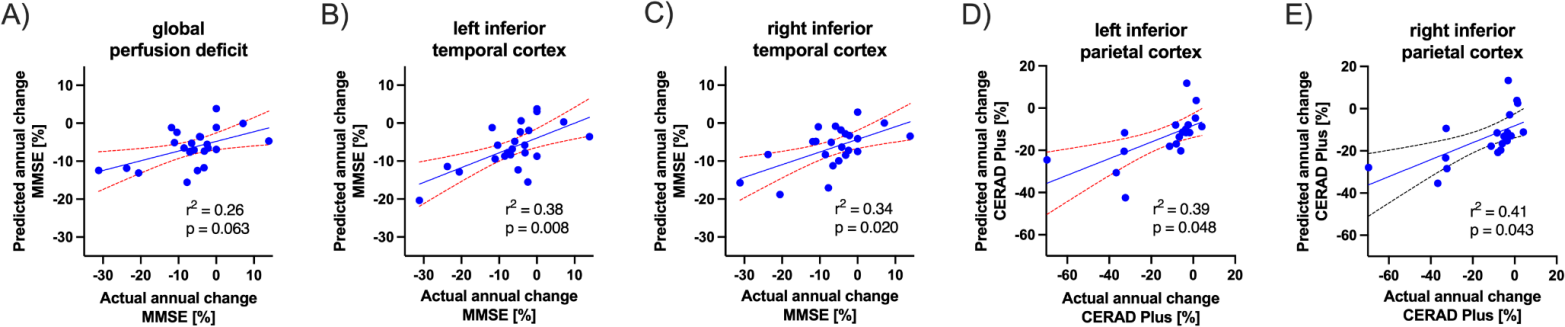
Multilinear regression of perfusion deficit and annual percentage decline of test results in neuropsychological testing (MMSE and CERAD) correcting for age, gender, and years of education

For 23 patients, follow-up data of neuropsychological testing was available with a median follow-up time of 524 days (interquartile range: 358–1,011 days). Multiple linear regression analysis corrected for age, sex, and education revealed that perfusion deficits in the temporal lobes predicted future annual decline of MMSE scores (r^2^ = 0.29; *p* = 0.037), while there was only a trend for global perfusion deficits (r^2^ = 0.26, *p* = 0.063, Fig. 5A). The strongest association was detected for the left inferior temporal cortex (r^2^ = 0.38; *p* = 0.008). The perfusion deficit of the inferior parietal lobe showed a good prediction of future annual decline of CERAD-Plus sum scores (r^2^ = 0.39–0.41; *p* = 0.043–0.048). Results are displayed in Fig. 5. Voxel-weighted mean z-scores of regions III/IV showed a significant association, voxel-weighted mean z-scores of regions V/VI a trend towards an association with annual percentage decline of MMSE scores (r^2^ = 0.15; *p* = 0.033 and r^2^ = 0.12; *p* = 0.054). There was no significant difference in percentage annual decline of patients grouped as stage^0^, stage^I-II+^, stage^I-IV+^ and stage^I-VI+^. While 21 patients showed decreasing MMSE scores, there were 2 patients with an annual elevation in MMSE test scores (+ 7.0% and + 13.9%). There were three patients with a small annual increase of CERAD Plus sum score (+ 1.1%, + 1.5% and + 7.1%).

## Discussion

In our study, we were able to demonstrate an association of global and regional perfusion deficits respectively in early-phase β-amyloid-PET with current cognitive performance in MMSE testing and CERAD-plus testing and future cognitive decline, as well as the feasibility of perfusion based staging thereby indicating the utility of early-phase β-amyloid-PET as a surrogate for neurodegeneration severity.

Hypoperfused brain regions were highly correlated with those found to be impaired during neuropsychological testing. The strongest regional association with neuropsychological test results was detected in the left temporal lobe, encompassing multiple structures involved in memory processing and linguistic abilities, like the hippocampus in the mesial temporal lobe or the temporal pole [30]. A strong agreement of left temporal lobe hypoperfusion with clinical testing in AD has already been shown in various MRI and FDG-PET/CT studies [2, 31–33]. Clinical testing with MMSE also showed an association with perfusion deficits in the bilateral inferior and posteromedial parietal lobe [34–36]. In comparison to MMSE testing, we noted a stronger correlation between CERAD-Plus sum scores and frontal perfusion impairments. The difference can be explained by the CERAD-Plus battery’s increased focus on executive skills and semantic evaluations (e.g., in the subtests of semantic verbal fluency and the Boston naming test) that activate the frontal lobe [22]. Results in TMT A were linked not only to temporal perfusion deficits, but also to parietal and occipital perfusion abnormalities, attributable to the parietal lobes’ established function in visuospatial orientation and visuomotor control [37] alongside the occipital lobes’ known role in visual processing [38, 39]. Not only regional, but also the global perfusion deficit demonstrated strong associations with neuropsychological testing using MMSE and CERAD-Plus test battery. This agrees with a previous study demonstrating the efficacy of early-phase β-amyloid-PET in distinguishing the degree of hypoperfusion between Alzheimer’s disease patients and healthy controls, and showing a significant correlation between the hypoperfusion in AD-related regions and MMSE scores [11]. Our results also corroborate previous research consistently showing strong associations between MRI assessed whole brain volume and disease severity and progression in AD [40–44].

Overall, disease severity as assessed by neuropsychological testing showed a higher association with left hemispheric perfusion deficits confirming previous results describing a left-leaning pattern of neurodegeneration and β-amyloid pathology in AD [31, 45–48].

In our study, regional perfusion deficits showed a better correlation than the summarized perfusion deficits in predefined regions that were oriented to the regions that are affected by tau-fibrils in histopathologic Braak-staging. This may be due to regions known to have a high tau burden, but not consistently showing a strong hypoperfusion in earlier studies, such as the temporal region [49].

In our study, early-phase β-amyloid-PET-based staging effectively classified the majority of patients across distinct clinical severities of dementia. Despite the exclusion of atypical patients, 19.5% of patients were classified as stage^atypical+^, a result that was unexpected. A similar percentage of Alzheimer’s patients have an aberrant distribution of tau fibrils, yet there are known regions, such as the temporal lobe, where tau deposition and hypoperfusion do not correspond [49].

In our study, the percentage decline of clinical test scores was associated with temporal and parietal perfusion impairment. The minor improvement in test scores observed in a limited number of patients is likely attributable to daily fluctuations in cognitive performance. In line with our data, previous MRI perfusion and FDG-PET studies have extensively validated the association of regional perfusion impairment and cognitive decline with the best regional associations being reported in the hippocampus, the entorhinal cortex, and the posterior cingulate cortex [3, 50–52]. Also, frontal and parietal perfusion deficits have been described to significantly correlate with cognitive decline as disease progresses [53–56]. In conclusion, β-amyloid-PET may not only be useful for diagnostic purposes but also provides a tool to estimate patient-specific risk for cognitive decline and clinical progression in AD.

Until recently, therapeutic options available to patients with AD were confined to symptomatic treatments. However, antibodies targeting β-amyloid plaques have meanwhile received FDA approval and further promising targeted therapies aiming to mitigate cognitive decline are currently being developed. The various pathomechanisms of these drugs will eventually allow individualized therapeutic approaches. In order to evaluate therapeutic agents, biomarkers that correlate with disease severity and progression are needed. In our study, regional and global perfusion deficits measured by early-phase β-amyloid-PET were associated with current neuropsychological test results and clinical progression underscoring their potential for usage in clinical trials for assessing and staging neurodegeneration severity in AD. Various studies demonstrated a strong agreement between regional perfusion deficits in early-phase β-amyloid-, tau-, and dopamine-PET, regional perfusion deficits in perfusion MRI and hypometabolism assessed by FDG-PET [6, 10, 57–60]. Recently, the European intersocietal recommendations for the biomarker-based diagnosis of neurocognitive disorders were published [61]. According to the proposed diagnostic workflow, FDG-PET should be used in the workup of patients with a causal hypothesis of suspected AD, frontotemporal lobar degeneration or suspected motor tauopathy to assess the pattern of neuronal injury [61]. Depending on the suspected clinical diagnosis and already measured biomakers, an additional assessment of CSF biomarkers is required [61]. The detected associations of this study underline the possibility to detect neuronal injury by early-phase β-amyloid-PET in parallel to assessing amyloid burden. A dual-phase acquisition protocol of β-amyloid-PET may therefore eliminate the need to perform further examinations. Also, in clinically unclear cases, the concurrent measurement of two biomarker categories in a one-stop-shop examination may be time-saving and cost-effective. However, especially in younger patients, atypical clinical presentations, or rapid progression of dementia, CSF examination remains essential to rule out potential differential diagnoses. Furthermore, early-phase β-amyloid-PET could represent an alternative to FDG-PET imaging regarding the prediction of cognitive decline. However, head-to-head studies will be needed to compare the predictive accuracy of early-phase β-amyloid-PET and FDG- and tau-PET, respectively.

Several limitations apply to our study. We acknowledge the comparatively low number of patients with follow-up data. Larger prospective studies will be needed to verify the detected association of early-phase β-amyloid-PET with future cognitive decline. Furthermore, PET-based staging followed the assumption that perfusion deficits are highest in regions with highest tau load. Despite many regions overlapping between tau deposition and neurodegeneration, the temporal decoupling and some different vulnerabilities make the patterns not completely fitting. Previous studies even suggested that hypoperfusion can precede tau deposition [62]. Our study lacks a correlation with histopathological data or tau-PET to confirm pathological tau fibrils in brain regions with reduced perfusion. Reduced regional perfusion is non-specific and may be related to other underlying pathologies such as LATE-pathology, synuclein, ischemic lesions or white matter changes. Therefore, further studies will be needed to evaluate the association of early-phase β-amyloid-PET-based staging with histopathologically confirmed Braak-stages. Also, there are currently no established cut-offs for the definition of pathological hypoperfusion in early-phase β-amyloid-PET. In our study, we used a modified AAL-atlas with a parcellation into 47 regions. While the temporomesial lobe encompassing several important structures of the memory system was not divided further into subregions, this approach is close to daily patient evaluation and may therefore be suitable for clinical routine. Moreover, in contrast to the diagnostic evaluation of the cerebral hypometabolism in FDG-PET, commercial softwares don’t provide a built-in normal cohort for z-score evaluation of early-phase amyloid-PET yet. However, the assessment of perfusion deficits by early-phase β-amyloid-PET is very robust and easy to include in the acquisition process and postprocessing workflow. Normal-cohort data could readily be implemented in the future, if early-phase β-amyloid-PET will further be established as a marker of neuronal injury.

Keeping these limitations in mind, our data reveal that global and regional perfusion deficits on early-phase β-amyloid-PET can serve as an objective index of clinical testing and may act as a prognostic marker of future cognitive decline in AD. The possibility to assess two biomarker categories in a one-stop-shop examination may reduce the need for additional diagnostic procedures assessing β-amyloid and neurodegeneration in the diagnostic work-up of patients with suspected AD.

## Electronic supplementary material

Below is the link to the electronic supplementary material.

Supplementary Material 1

## Data Availability

The datasets generated during and/or analysed during the current study are available from the corresponding author on reasonable request.
