# Supplementary material for: Correlation of early-phase β-amyloid positron-emission-tomography and neuropsychological testing in patients with Alzheimer’s disease
Source: Eur J Nucl Med Mol Imaging. 2025 Feb 28;52(8):2918–28. doi: 10.1007/s00259-025-07175-5 (PMC12162376; doi:10.1007/s00259-025-07175-5)
Supplement: Supplementary file 1 — Supplementary Material 1 [file 259_2025_7175_MOESM1_ESM.docx]

**Supplementary Table 1. Post-hoc subtests (Tukey’s multiple comparisons test) to compare age, gender, years of education, MMSE and CERAD Plus sumscore and subtests of patients with different MMSE scores**

| Age |  |  |  |  |
| --- | --- | --- | --- | --- |
| Tukey's multiple comparisons test | Mean 1 | Mean 2 | Adjusted P Value | Summary |
| MMSE 28 - 30 vs.  MMSE 24 - 27 | 70.3 | 71.8 | 0.927 | ns |
| MMSE 28 - 30 vs. MMSE 18 - 23 | 70.3 | 73.1 | 0.721 | ns |
| MMSE 28 - 30 vs. MMSE 10 - 17 | 70.3 | 71 | 0.996 | ns |
| MMSE 24 - 27 vs. MMSE 18 - 23 | 71.8 | 73.1 | 0.928 | ns |
| MMSE 24 - 27 vs. MMSE 10 - 17 | 71.8 | 71 | 0.991 | ns |
| MMSE 18 - 23 vs. MMSE 10 - 17 | 73.1 | 71 | 0.885 | ns |
|  |  |  |  |  |
| Education |  |  |  |  |
| Tukey's multiple comparisons test | Mean 1 | Mean 2 | Adjusted P Value | Summary |
| MMSE 28 - 30 vs.  MMSE 24 - 27 | 14.6 | 13.2 | 0.529 | ns |
| MMSE 28 - 30 vs. MMSE 18 - 23 | 14.6 | 12.6 | 0.294 | ns |
| MMSE 28 - 30 vs. MMSE 10 - 17 | 14.6 | 13.1 | 0.667 | ns |
| MMSE 24 - 27 vs. MMSE 18 - 23 | 13.2 | 12.6 | 0.909 | ns |
| MMSE 24 - 27 vs. MMSE 10 - 17 | 13.2 | 13.1 | 1 | ns |
| MMSE 18 - 23 vs. MMSE 10 - 17 | 12.6 | 13.1 | 0.977 | ns |
|  |  |  |  |  |
| MMSE |  |  |  |  |
| Tukey's multiple comparisons test | Mean 1 | Mean 2 | Adjusted P Value | Summary |
| MMSE 28 - 30 vs.  MMSE 24 - 27 | 28.79 | 25.17 | <0.0001 | **** |
| MMSE 28 - 30 vs. MMSE 18 - 23 | 28.79 | 21.63 | <0.0001 | **** |
| MMSE 28 - 30 vs. MMSE 10 - 17 | 28.79 | 13.64 | <0.0001 | **** |
| MMSE 24 - 27 vs. MMSE 18 - 23 | 25.17 | 21.63 | <0.0001 | **** |
| MMSE 24 - 27 vs. MMSE 10 - 17 | 25.17 | 13.64 | <0.0001 | **** |
| MMSE 18 - 23 vs. MMSE 10 - 17 | 21.63 | 13.64 | <0.0001 | **** |
|  |  |  |  |  |
| CERAD sum score |  |  |  |  |
| Tukey's multiple comparisons test | Mean 1 | Mean 2 | Adjusted P Value | Summary |
| MMSE 28 - 30 vs.  MMSE 24 - 27 | 68.86 | 61.86 | 0.135 | ns |
| MMSE 28 - 30 vs. MMSE 18 - 23 | 68.86 | 52.28 | <0.0001 | **** |
| MMSE 28 - 30 vs. MMSE 10 - 17 | 68.86 | 43.67 | <0.0001 | **** |
| MMSE 24 - 27 vs. MMSE 18 - 23 | 61.86 | 52.28 | 0.005 | ** |
| MMSE 24 - 27 vs. MMSE 10 - 17 | 61.86 | 43.67 | <0.0001 | **** |
| MMSE 18 - 23 vs. MMSE 10 - 17 | 52.28 | 43.67 | 0.111 | ns |
|  |  |  |  |  |
| word list learning |  |  |  |  |
| Tukey's multiple comparisons test | Mean 1 | Mean 2 | Adjusted P Value | Summary |
| MMSE 28 - 30 vs.  MMSE 24 - 27 | 14.5 | 11.83 | 0.294 | ns |
| MMSE 28 - 30 vs. MMSE 18 - 23 | 14.5 | 11.11 | 0.177 | ns |
| MMSE 28 - 30 vs. MMSE 10 - 17 | 14.5 | 4.7 | <0.0001 | **** |
| MMSE 24 - 27 vs. MMSE 18 - 23 | 11.83 | 11.11 | 0.947 | ns |
| MMSE 24 - 27 vs. MMSE 10 - 17 | 11.83 | 4.7 | 3E-04 | *** |
| MMSE 18 - 23 vs. MMSE 10 - 17 | 11.11 | 4.7 | 0.003 | ** |
|  |  |  |  |  |
| word list recall |  |  |  |  |
| Tukey's multiple comparisons test | Mean 1 | Mean 2 | Adjusted P Value | Summary |
| MMSE 28 - 30 vs.  MMSE 24 - 27 | 5.21 | 3.06 | 7E-04 | *** |
| MMSE 28 - 30 vs. MMSE 18 - 23 | 5.21 | 1.84 | <0.0001 | **** |
| MMSE 28 - 30 vs. MMSE 10 - 17 | 5.21 | 0.5 | <0.0001 | **** |
| MMSE 24 - 27 vs. MMSE 18 - 23 | 3.06 | 1.84 | 0.048 | * |
| MMSE 24 - 27 vs. MMSE 10 - 17 | 3.06 | 0.5 | 2E-04 | *** |
| MMSE 18 - 23 vs. MMSE 10 - 17 | 1.84 | 0.5 | 0.149 | ns |
|  |  |  |  |  |
| word list intrusions |  |  |  |  |
| Tukey's multiple comparisons test | Mean 1 | Mean 2 | Adjusted P Value | Summary |
| MMSE 28 - 30 vs.  MMSE 24 - 27 | 1.38 | 2.71 | 0.652 | ns |
| MMSE 28 - 30 vs. MMSE 18 - 23 | 1.38 | 3.83 | 0.214 | ns |
| MMSE 28 - 30 vs. MMSE 10 - 17 | 1.38 | 1.71 | 0.996 | ns |
| MMSE 24 - 27 vs. MMSE 18 - 23 | 2.71 | 3.83 | 0.677 | ns |
| MMSE 24 - 27 vs. MMSE 10 - 17 | 2.71 | 1.71 | 0.855 | ns |
| MMSE 18 - 23 vs. MMSE 10 - 17 | 3.83 | 1.71 | 0.403 | ns |
|  |  |  |  |  |
| word list recognition |  |  |  |  |
| Tukey's multiple comparisons test | Mean 1 | Mean 2 | Adjusted P Value | Summary |
| MMSE 28 - 30 vs.  MMSE 24 - 27 | 9.5 | 8.43 | 0.306 | ns |
| MMSE 28 - 30 vs. MMSE 18 - 23 | 9.5 | 8.05 | 0.143 | ns |
| MMSE 28 - 30 vs. MMSE 10 - 17 | 9.5 | 7.86 | 0.164 | ns |
| MMSE 24 - 27 vs. MMSE 18 - 23 | 8.43 | 8.05 | 0.892 | ns |
| MMSE 24 - 27 vs. MMSE 10 - 17 | 8.43 | 7.86 | 0.83 | ns |
| MMSE 18 - 23 vs. MMSE 10 - 17 | 8.05 | 7.86 | 0.994 | ns |
|  |  |  |  |  |
| word list discriminability |  |  |  |  |
| Tukey's multiple comparisons test | Mean 1 | Mean 2 | Adjusted P Value | Summary |
| MMSE 28 - 30 vs.  MMSE 24 - 27 | 7.43 | 6.2 | 0.72 | ns |
| MMSE 28 - 30 vs. MMSE 18 - 23 | 7.43 | 7.28 | 0.999 | ns |
| MMSE 28 - 30 vs. MMSE 10 - 17 | 7.43 | 5.33 | 0.501 | ns |
| MMSE 24 - 27 vs. MMSE 18 - 23 | 6.2 | 7.28 | 0.715 | ns |
| MMSE 24 - 27 vs. MMSE 10 - 17 | 6.2 | 5.33 | 0.904 | ns |
| MMSE 18 - 23 vs. MMSE 10 - 17 | 7.28 | 5.33 | 0.496 | ns |
|  |  |  |  |  |
| word list recognition |  |  |  |  |
| Tukey's multiple comparisons test | Mean 1 | Mean 2 | Adjusted P Value | Summary |
| MMSE 28 - 30 vs.  MMSE 24 - 27 | 10.92 | 9.74 | 0.37 | ns |
| MMSE 28 - 30 vs. MMSE 18 - 23 | 10.92 | 7.72 | 7E-04 | *** |
| MMSE 28 - 30 vs. MMSE 10 - 17 | 10.92 | 7.43 | 0.002 | ** |
| MMSE 24 - 27 vs. MMSE 18 - 23 | 9.74 | 7.72 | 0.011 | * |
| MMSE 24 - 27 vs. MMSE 10 - 17 | 9.74 | 7.43 | 0.024 | * |
| MMSE 18 - 23 vs. MMSE 10 - 17 | 7.72 | 7.43 | 0.987 | ns |
|  |  |  |  |  |
| figure recall |  |  |  |  |
| Tukey's multiple comparisons test | Mean 1 | Mean 2 | Adjusted P Value | Summary |
| MMSE 28 - 30 vs.  MMSE 24 - 27 | 7.23 | 4.69 | 0.007 | ** |
| MMSE 28 - 30 vs. MMSE 18 - 23 | 7.23 | 2.94 | <0.0001 | **** |
| MMSE 28 - 30 vs. MMSE 10 - 17 | 7.23 | 0.71 | <0.0001 | **** |
| MMSE 24 - 27 vs. MMSE 18 - 23 | 4.69 | 2.94 | 0.049 | * |
| MMSE 24 - 27 vs. MMSE 10 - 17 | 4.69 | 0.71 | <0.0001 | **** |
| MMSE 18 - 23 vs. MMSE 10 - 17 | 2.94 | 0.71 | 0.072 | ns |
|  |  |  |  |  |
| semantic verbal fluency |  |  |  |  |
| Tukey's multiple comparisons test | Mean 1 | Mean 2 | Adjusted P Value | Summary |
| MMSE 28 - 30 vs.  MMSE 24 - 27 | 14.5 | 15.49 | 0.908 | ns |
| MMSE 28 - 30 vs. MMSE 18 - 23 | 14.5 | 12.33 | 0.538 | ns |
| MMSE 28 - 30 vs. MMSE 10 - 17 | 14.5 | 9 | 0.023 | * |
| MMSE 24 - 27 vs. MMSE 18 - 23 | 15.49 | 12.33 | 0.075 | ns |
| MMSE 24 - 27 vs. MMSE 10 - 17 | 15.49 | 9 | 7E-04 | *** |
| MMSE 18 - 23 vs. MMSE 10 - 17 | 12.33 | 9 | 0.231 | ns |
|  |  |  |  |  |
| phonematic verbal fluency |  |  |  |  |
| Tukey's multiple comparisons test | Mean 1 | Mean 2 | Adjusted P Value | Summary |
| MMSE 28 - 30 vs.  MMSE 24 - 27 | 11.29 | 10.29 | 0.869 | ns |
| MMSE 28 - 30 vs. MMSE 18 - 23 | 11.29 | 8.72 | 0.28 | ns |
| MMSE 28 - 30 vs. MMSE 10 - 17 | 11.29 | 6.71 | 0.036 | * |
| MMSE 24 - 27 vs. MMSE 18 - 23 | 10.29 | 8.72 | 0.513 | ns |
| MMSE 24 - 27 vs. MMSE 10 - 17 | 10.29 | 6.71 | 0.066 | ns |
| MMSE 18 - 23 vs. MMSE 10 - 17 | 8.72 | 6.71 | 0.562 | ns |
|  |  |  |  |  |
| Boston naming test |  |  |  |  |
| Tukey's multiple comparisons test | Mean 1 | Mean 2 | Adjusted P Value | Summary |
| MMSE 28 - 30 vs.  MMSE 24 - 27 | 13.64 | 13.43 | 0.991 | ns |
| MMSE 28 - 30 vs. MMSE 18 - 23 | 13.64 | 12.32 | 0.349 | ns |
| MMSE 28 - 30 vs. MMSE 10 - 17 | 13.64 | 10.2 | 0.002 | ** |
| MMSE 24 - 27 vs. MMSE 18 - 23 | 13.43 | 12.32 | 0.301 | ns |
| MMSE 24 - 27 vs. MMSE 10 - 17 | 13.43 | 10.2 | 6E-04 | *** |
| MMSE 18 - 23 vs. MMSE 10 - 17 | 12.32 | 10.2 | 0.071 | ns |
|  |  |  |  |  |
| TMT A |  |  |  |  |
| Tukey's multiple comparisons test | Mean 1 | Mean 2 | Adjusted P Value | Summary |
| MMSE 28 - 30 vs.  MMSE 24 - 27 | 57.64 | 74.53 | 0.614 | ns |
| MMSE 28 - 30 vs. MMSE 18 - 23 | 57.64 | 84.2 | 0.302 | ns |
| MMSE 28 - 30 vs. MMSE 10 - 17 | 57.64 | 121.8 | 0.003 | ** |
| MMSE 24 - 27 vs. MMSE 18 - 23 | 74.53 | 84.2 | 0.853 | ns |
| MMSE 24 - 27 vs. MMSE 10 - 17 | 74.53 | 121.8 | 0.013 | * |
| MMSE 18 - 23 vs. MMSE 10 - 17 | 84.2 | 121.8 | 0.106 | ns |

**Supplementary Table 2. Regional perfusion deficit (z-scores) in patients with different grade of clinical severity of dementia based on MMSE scores.**

|  | MMSE 28-30 | | MMSE 24 - 27 | | MMSE 18 - 23 | | MMSE 10 - 17 | |
| --- | --- | --- | --- | --- | --- | --- | --- | --- |
|  | **mean** | **std** | **mean** | **std** | **mean** | **Std** | **mean** | **std** |
| L Precentral Cortex | -0.70 | 0.97 | -0.67 | 0.96 | -1.21 | 0.82 | -0.53 | 1.11 |
| R Precentral Cortex | -0.34 | 0.91 | -0.51 | 1.06 | -1.26 | 1.93 | -0.60 | 1.95 |
| L Orbitofrontal Cortex | -1.78 | 1.37 | -1.20 | 0.97 | -1.52 | 0.98 | -1.64 | 1.38 |
| R Orbitofrontal Cortex | -1.54 | 1.16 | -1.11 | 0.80 | -1.79 | 1.62 | -1.41 | 1.43 |
| L Frontal Lateral | -1.64 | 1.35 | -1.75 | 1.06 | -2.36 | 1.20 | -2.45 | 1.60 |
| R Frontal Lateral | -1.55 | 0.96 | -1.74 | 1.04 | -2.68 | 2.11 | -2.42 | 1.99 |
| L Frontal Medial | -1.48 | 1.29 | -1.38 | 0.99 | -1.96 | 1.46 | -1.50 | 1.51 |
| R Frontal Medial | -1.32 | 1.31 | -1.50 | 1.03 | -2.10 | 1.36 | -1.96 | 1.63 |
| L Insula | -0.55 | 1.64 | -0.94 | 1.24 | -0.92 | 1.20 | -1.71 | 1.93 |
| R Insula | -0.23 | 1.72 | -0.30 | 1.39 | -1.08 | 2.95 | -0.77 | 1.82 |
| L Anterior Cingulate | -0.66 | 1.42 | -0.73 | 1.33 | -0.98 | 1.74 | -1.02 | 2.16 |
| R Anterior Cingulate | -0.82 | 1.18 | -1.12 | 1.17 | -1.51 | 1.64 | -1.52 | 1.66 |
| L Posterior Cingulate | -1.18 | 0.88 | -1.74 | 1.36 | -1.80 | 1.09 | -2.39 | 1.38 |
| R Posterior Cingulate | -1.50 | 0.72 | -1.85 | 1.21 | -1.98 | 0.94 | -2.55 | 1.11 |
| L Mesio-Temporal Lobe | -0.39 | 1.70 | -0.52 | 1.21 | -0.69 | 1.09 | -1.66 | 2.08 |
| R Mesio-Temporal Lobe | 0.02 | 1.55 | -0.13 | 1.34 | -0.71 | 1.59 | -1.22 | 1.87 |
| L Mesio-Occipital Lobe | -0.60 | 1.02 | -0.83 | 1.26 | -0.95 | 1.50 | -1.46 | 2.14 |
| R Mesio-Occipital Lobe | -0.96 | 1.03 | -1.17 | 1.71 | -1.38 | 2.00 | -2.15 | 2.46 |
| L Occipital Cortex | -2.25 | 1.17 | -2.15 | 1.64 | -2.63 | 1.79 | -3.00 | 2.07 |
| R Occipital Cortex | -1.35 | 1.04 | -1.58 | 1.51 | -1.92 | 1.45 | -2.44 | 2.32 |
| L Postcentral Cortex | -1.16 | 0.55 | -1.01 | 1.01 | -1.22 | 0.93 | -1.61 | 1.89 |
| R Postcentral Cortex | -1.17 | 0.59 | -1.28 | 1.24 | -1.70 | 2.10 | -1.51 | 1.64 |
| L Sup Parietal Cortex | -0.92 | 0.90 | -1.28 | 1.59 | -1.43 | 1.49 | -1.71 | 1.06 |
| R Sup Parietal Cortex | -0.67 | 0.80 | -0.89 | 1.08 | -1.38 | 1.01 | -1.23 | 0.69 |
| L Inf Parietal Cortex | -2.15 | 1.04 | -2.58 | 1.46 | -3.21 | 1.13 | -3.06 | 1.31 |
| R Inf Parietal Cortex | -2.33 | 1.04 | -2.70 | 1.51 | -3.59 | 1.60 | -3.27 | 1.84 |
| L Precuneus | -2.53 | 1.37 | -2.94 | 1.50 | -3.43 | 1.64 | -4.17 | 1.40 |
| R Precuneus | -2.56 | 1.34 | -2.93 | 1.55 | -3.62 | 1.69 | -3.98 | 1.47 |
| L Caudate | -1.83 | 1.33 | -2.06 | 1.33 | -2.28 | 1.18 | -2.62 | 1.47 |
| R Caudate | -1.85 | 1.13 | -2.10 | 1.28 | -2.48 | 1.09 | -2.44 | 1.37 |
| L Putamen | 0.80 | 1.32 | 0.84 | 1.07 | 1.24 | 1.04 | 1.03 | 1.98 |
| R Putamen | 0.84 | 1.14 | 0.94 | 0.98 | 0.43 | 3.90 | 1.28 | 1.57 |
| L Pallidum | 0.08 | 0.91 | 0.43 | 1.26 | 0.62 | 1.13 | 0.44 | 1.89 |
| R Pallidum | 0.81 | 0.78 | 1.06 | 1.14 | 0.57 | 2.37 | 1.06 | 1.38 |
| L Thalamus | 1.00 | 1.97 | 0.93 | 2.11 | 0.68 | 1.61 | -0.07 | 2.81 |
| R Thalamus | -0.03 | 2.00 | -0.17 | 1.89 | -0.99 | 2.36 | -0.82 | 2.17 |
| L Sup Temporal Cortex | -1.34 | 0.95 | -1.49 | 1.08 | -1.90 | 1.31 | -2.63 | 1.78 |
| R Sup Temporal Cortex | -1.59 | 0.75 | -1.75 | 1.06 | -2.46 | 2.56 | -2.58 | 2.44 |
| L Mid Temporal Cortex | -1.83 | 1.21 | -1.99 | 1.74 | -2.99 | 2.06 | -4.02 | 3.11 |
| R Mid Temporal Cortex | -1.83 | 1.28 | -1.78 | 1.63 | -2.73 | 1.93 | -3.15 | 3.05 |
| L Inf Temporal Cortex | -1.21 | 1.15 | -1.38 | 1.90 | -2.23 | 2.04 | -3.29 | 3.42 |
| R Inf Temporal Coretex | -0.85 | 1.00 | -1.05 | 1.79 | -2.09 | 1.78 | -2.49 | 3.22 |
| L Temporal Pole | -0.83 | 0.93 | -0.98 | 1.07 | -1.27 | 1.16 | -1.66 | 1.47 |
| R Temporal Pole | -0.29 | 0.89 | -0.68 | 1.09 | -1.10 | 1.75 | -1.15 | 1.84 |
| L Cerebellum | 1.87 | 0.78 | 2.04 | 0.76 | 1.59 | 1.30 | 1.75 | 1.14 |
| R Cerebellum | 1.55 | 0.62 | 1.70 | 0.79 | 1.61 | 0.68 | 1.38 | 1.18 |
| Vermis | 0.76 | 0.42 | 0.68 | 0.73 | 0.79 | 0.79 | 1.09 | 1.24 |

**Supplementary table 3 posthoc subtest (Tukey’s multiple comparisons test) to compare hypoperfusion in region III/IV of patients with different MMSE score groups**

| **Table 3 region III/IV** |  |  |  |  |
| --- | --- | --- | --- | --- |
| Tukey's multiple comparisons test | Mean 1 | Mean 2 | Adjusted P Value | Summary |
| MMSE 28 - 30 vs.  MMSE 24 - 27 | -1.011 | -1.195 | 0.96 | ns |
| MMSE 28 - 30 vs. MMSE 18 - 23 | -1.011 | -1.741 | 0.296 | ns |
| MMSE 28 - 30 vs. MMSE 10 - 17 | -1.011 | -2.29 | 0.041 | * |
| MMSE 24 - 27 vs. MMSE 18 - 23 | -1.195 | -1.741 | 0.364 | ns |
| MMSE 24 - 27 vs. MMSE 10 - 17 | -1.195 | -2.29 | 0.041 | * |
| MMSE 18 - 23 vs. MMSE 10 - 17 | -1.741 | -2.29 | 0.606 | ns |
